# Host-Parasite Interactions and Population Dynamics of Rock Ptarmigan

**DOI:** 10.1371/journal.pone.0165293

**Published:** 2016-11-21

**Authors:** Ute Stenkewitz, Ólafur K. Nielsen, Karl Skírnisson, Gunnar Stefánsson

**Affiliations:** 1 Faculty of Life and Environmental Sciences, University of Iceland, Askja, Reykjavík, Iceland; 2 Icelandic Institute of Natural History, Garðabær, Iceland; 3 Institute for Experimental Pathology, University of Iceland, Keldur, Reykjavík, Iceland; 4 Science Institute, University of Iceland, Reykjavík, Iceland; University of Alberta, CANADA

## Abstract

Populations of rock ptarmigan (*Lagopus muta*) in Iceland fluctuate in multiannual cycles with peak numbers c. every 10 years. We studied the ptarmigan-parasite community and how parasites relate to ptarmigan age, body condition, and population density. We collected 632 ptarmigan in northeast Iceland in early October from 2006 to 2012; 630 (99.7%) were infected with at least one parasite species, 616 (98%) with ectoparasites, and 536 (85%) with endoparasites. We analysed indices for the combined parasite community (16 species) and known pathogenic parasites, two coccidian protozoans *Eimeria muta* and *Eimeria rjupa*, two nematodes *Capillaria caudinflata* and *Trichostrongylus tenuis*, one chewing louse *Amyrsidea lagopi*, and one skin mite *Metamicrolichus islandicus*. Juveniles overall had more ectoparasites than adults, but endoparasite levels were similar in both groups. Ptarmigan population density was associated with endoparasites, and in particular prevalence of the coccidian parasite *Eimeria muta*. Annual aggregation level of this eimerid fluctuated inversely with prevalence, with lows at prevalence peak and vice versa. Both prevalence and aggregation of *E*. *muta* tracked ptarmigan population density with a 1.5 year time lag. The time lag could be explained by the host specificity of this eimerid, host density dependent shedding of oocysts, and their persistence in the environment from one year to the next. Ptarmigan body condition was negatively associated with *E*. *muta* prevalence, an indication of their pathogenicity, and this eimerid was also positively associated with ptarmigan mortality and marginally inversely with fecundity. There were also significant associations between fecundity and chewing louse *Amyrsidea lagopi* prevalence (negative), excess juvenile mortality and nematode *Capillaria caudinflata* prevalence (positive), and adult mortality and skin mite *Metamicrolichus islandicus* prevalence (negative). Though this study is correlational, it provides strong evidence that *E*. *muta* through time-lag in prevalence with respect to host population size and by showing significant relations with host body condition, mortality, and fecundity could destabilize ptarmigan population dynamics in Iceland.

## Introduction

Parasite communities of wildlife species have rarely been studied over an extended time period [1]. Detailed studies can provide insight into many aspects of parasite ecology, the relationships between host and parasites, and adaptations resulting from this intimate network [1] [2] [3]. Parasites can influence population dynamics by affecting host body condition, fecundity, and survival, and host-parasite interactions are known to be one of the driving forces of multiannual cycles in animal populations (e.g., [4] [5] [6] [7] [8] [9]). Cycles have been reported in a variety of herbivorous species in northern latitudes including moths, hares, lemmings, voles, and grouse [9] [10], and are well documented for species in the genus *Lagopus*. The parasitic nematode *Trichostrongylus tenuis* is thought to be one of the agents driving the red grouse *Lagopus lagopus scoticus* cycle in Scotland [11]. Parasites of willow ptarmigan (*L*. *l*. *lagopus*) in Norway, have been reported to affect demographic parameters including body condition and breeding success of the host, and were negatively associated with changes in numbers of the ptarmigan population [12] [13].

Some rock ptarmigan (*Lagopus muta*) populations show multiannual cycles [13] [14] [15] [16]. Traditionally, the ptarmigan population in Iceland has cycled with a 10–12 year period [15], but recently this has changed and the periods have become shorter [17]. The gyrfalcon (*Falco rusticolus*) and the rock ptarmigan in Iceland have a coupled predator-prey cycle and the falcon is thought to be an agent driving the ptarmigan cycle [18] [19]. However, other researchers have demonstrated the importance of studying the effects of several different factors on the cyclic behaviour of a population (e.g., [20] [21] [22]). Parasites have the potential to effect host survival by making birds more prone to predation, weakening body condition, or changing host behaviour [12] [23] [24].

The parasite fauna of rock ptarmigan in Iceland has been described and 17 species have been reported [25] [26]. In this paper, we want to examine the potential role of the parasites on the population dynamics of rock ptarmigan. We do this by investigating the relationship between ptarmigan density, body condition, mortality, fecundity, and measures of parasite abundance and aggregation over a period of 7 years (2006–2012). We look at the combined parasite community and focus on six known pathogenic parasite species, namely *Eimeria muta* and *Eimeria rjupa*, intestinal microparasites that can cause coccidiosis [27], *Capillaria caudinflata*, a helminth known to cause capillariasis in Icelandic ptarmigan [28], *Trichostrongylus tenuis*, a helminth known to cause trichostrongylosis in grouse (also known as grouse disease) [3] [29], *Metamicrolichus islandicus*, a skin mite that can cause mange (pers. obs.), and *Amyrsidea lagopi*, an amblyceran chewing louse that can cause feather damage [30]. The Anderson and May model [4] [31] suggests that time delays in parasite abundance in relation to host population size, reduced aggregation of parasites during periods of high abundance, and the influence of parasites on host mortality and fecundity are the three destabilizing qualities of parasite-host dynamics. So, if the parasite community or any of the parasites of the rock ptarmigan are of importance, we expect the prevalence of a species to track host density, but with a time-lag, be least aggregated during the parasite’s peak of prevalence, and show a direct relation with the host’s body condition, fecundity, and/or mortality.

## Methods

### Study Area

The study area is in northeast Iceland centred on Lake Mývatn (65°40’ N 17°00’ W). The general topography is flat with rolling hills rising from the coast to 400–500 m above sea level at the southern border, 70 km inland. This relief is broken by isolated mountains, the highest being Bláfjall, 1222 m above sea level. Two major glacial rivers border the study area, Skjálfandafljót in the west and Jökulsá á Fjöllum in the east. Heath vegetation characterizes the xeric uplands. Important heath plants include dwarf shrubs such as *Betula nana* and *Salix phylicifolia*, and many species belonging to the heather family (Ericaceae) including *Empetrum nigrum*. Also important are various species of grasses (Poaceae), sedges (Carex), mosses, and lichens. In summer the ptarmigan is common on heath and grassland habitats. Winter habitats include alpine areas, rough lava fields and *Betula pubescens* shrubs.

### Ptarmigan Body Condition and Parasites

#### Ptarmigan for parasite analyses

Birds used for this analysis were collected specifically for a long-term study on the relation between ptarmigan population change and ptarmigan’s health related parameters (i.e., Skírnisson et al. 2012, Stenkewitz et al. 2015) that has been authorised by the Icelandic Institute of Natural History under law 64/1994, chapter 4, article 7 (http://www.althingi.is/lagas/140a/1994064.html). To do all the sampling and analysis required for the study at large it was necessary to sacrifice birds. But it should be noted that the ptarmigan is very common in Iceland and a popular game bird and since 1995 between 40 and 160 thousand birds have been shot every year (www.ust.is).

The birds were collected by experienced hunters using shotguns in moorlands, lava fields and alpine areas west, east, and north of Lake Mývatn in the 1^st^ week of October, 2006–2012. We chose the first week of October as the reference point to: (a) account for seasonal changes in parasite measures and size of anatomical and physiological features of ptarmigan [3] [32] [33]; and (b) sample the ptarmigan population at the start of winter as winter survival defines population change [34] [35]. Ptarmigan are free-flying wild birds and hunters could not select individuals at random. Birds were collected by conventional walk-up hunting where they were shot sitting or flying when encountered. Hunters tagged each bird immediately after collection, and to avoid cross-contamination it was wrapped in absorbent paper and placed in a paper bag. Each bag was sealed by interfolding and stapling. Birds were cooled to 4°C and dissected within 3 days of collection.

The annual goal was to sample 100 birds, 40 adults and 60 juveniles. This was achieved in most years for juveniles (2006 60, 2007 60, 2008 57, 2009 59, 2010 60, 2011 60, 2012 60), but not adults (2006 31, 2007 20, 2008 25, 2009 19, 2010 40, 2011 41, 2012 40). We analyzed all adults caught each year, but juveniles were shot in excess and we selected individuals from those at random, but kept the sex ratio equal.

#### Age identification

We assigned the age of each bird based on pigmentation of the primaries [36] and in the laboratory this was confirmed during necropsy by inspecting for presence (juvenile) or absence (adult) of the bursa of Fabricius. We recognized two age classes: juveniles (about 3 months of age) and adults (about 15 months or older).

#### Body condition

We calculated a body condition index by regressing body mass on body size and using the residuals as the index. For body size, we took six external and internal morphometric measurements for each bird: (a) wing length, measured with a ruler from the carpal joint to the tip of the flattened and straightened wing to the nearest mm; (b) head + bill length, measured with calipers from the hindmost point of the head to the tip of the bill to the nearest 0.1 mm; (c) tarsus length, measured with calipers from the joint between tarsus and toes to the intertarsal joint to the nearest 0.1 mm; (d) tarsus and mid-toe length, measured with a ruler from the joint to the base of the central claw to the nearest mm; (e) sternum length, measured with calipers from the tip of the *Spina externa* along the center line to the *Margo caudalis* to the nearest 0.1 mm; and (f) sternum-coracoid length, measured with calipers from the center line of the *Margo caudalis* to the cranial end of the *Coracoideum* to the nearest 0.1 mm (anatomical terms follow [37]). These six body measures (a–f) were highly correlated with each other. Factor 1 from a principle component analysis (PCA) was used as an index of body size. This Factor explained 61.4% of the variance in the original variables and was highly related to them (loadings: wing = 0.831; head + bill = 0.833; tarsus = 0.528; tarsus + mid-toe = 0.647; sternum = 0.891; and sternum-coracoid = 0.899).

#### Collection and quantification of ectoparasites

We collected and quantified species of one hippoboscid (*Ornithomya chloropus*), three mallophagans (*Goniodes lagopi*, *Lagopoecus affinis*, *Amyrsidea lagopi*), one flea (*Ceratophyllus garei*), four astigmatan (*Tetraolichus lagopi*, *Strelkoviacarus holoaspis*, *Metamicrolichus islandicus*, *Myialges borealis*) and one prostigmatan (*Mironovia lagopus*) mite/s using procedures described by [25]. Mallophagans and mites were embedded in Hoyer’s medium [38] for later identification based on [39] (*G*. *lagopi*, *L*. *affinis*), [40] (*A*. *lagopi*), [41] (Astigmata), and [42] (*M*. *lagopus*). *O*. *chloropus* was identified following [43].

Scoring was used to quantify the abundance of the quill mite *M*. *lagopus*. We examined quills of seven feathers–the upper-wing primary coverts 4 and 5 and secondary flight feathers 3–7 (numbered distal to proximal). Each feather was scored: 0 = no mites, 1 ≤ 10 mites present, and 2 > 10 mites present. Scores were summed to derive a value for each individual.

#### Collection and quantification of endoparasites

We removed the lower gastrointestinal tract. The small intestine and large intestine were separated from the ceca, placed in a zip lock plastic bag, and stored at -20°C. Fecal material (1–2 g) was taken from the large intestine prior to freezing, or, if the large intestine was empty, the posterior part of the small intestine. The modified McMaster procedure described in [25] was applied to obtain quantitative values of oocysts of *Eimeria muta* and *E*. *rjupa*, and their identification was based on [44]. *Blastocystis* sp. were found, but not quantified.

The small intestine and ceca were examined for the presence of helminths (*Capillaria caudinflata*, *T*. *tenuis*, *Passerilepis serpentulus*) following [25], and identified following [45], [46], and [47] for the nematodes *C*. *caudinflata* and *T*. *tenuis*, and Alexander Galkin at the Russian Academy of Sciences, St. Petersburg for the cestode *P*. *serpentulus*.

#### Parasite measures

We examined the parasite community (i.e., all parasite species known for the Icelandic rock ptarmigan except *Blastocystis* sp. and *Mesocestoides canislagopodis*) for adult and juvenile hosts in all years of the study. For this, we used a comparative value for each bird consisting of the ranked parasite data where 1 was allotted to the lowest positive finding and midranks were used for ties [12]. The ranked values of each parasite species were summed depicting the abundance for endoparasites, ectoparasites, and all parasites for each bird. We further used parasite richness, the mean of the total number of parasite species of an individual host in each age group [48].

For the individual parasite species we used parasite prevalence and aggregation. Parasite prevalence was defined as the proportion of birds infected by a particular parasite species [48]. Parasite aggregation was illustrated by a measure that applies to the right skewed parasite distributions, the Discrepancy index *D* [49]. This is a dimensionless index that ranges between 0 and 1; the distribution becomes more aggregated as the value approaches 1.

### Ptarmigan Population and Demographics

We collected three sets of population data: (1) spring densities of territorial males, (2) age ratios in spring, and (3) age ratios in late summer. From those, measures of fecundity and mortality were derived.

#### Ptarmigan spring densities

Territorial male ptarmigan were counted on six plots each spring starting in 1981. The total size of these plots was 26.8 km^2^ (range 2.4–8.0 km^2^). Each plot was surveyed once during 10–24 May. The survey was conducted on foot by at least two observers in the early morning (05:00–10:00) or late afternoon (17:00–24:00). The locations of territorial males as well as ptarmigan kills were plotted on a map. A “kill” was the remains of a ptarmigan killed and eaten after arrival on the census plot in spring. The main cause of death was predation by gyrfalcon (84%) [50]. The “freshness” of the kill was based on the state of the feathers. The total number of males in spring is composed of the sum of the number of territorial males censused and killed. Not all kills could be sexed, so to estimate the proportion of males we used the sex ratio of ptarmigan killed by gyrfalcons in spring on the study area (73% males) [51]. [52] provides a detailed description of the census plots and methods. The ptarmigan population abundance index used was the annual mean density of males on these six plots and covers the years 2004–2013.

#### Age ratios in Spring

Fully grown ptarmigan were aged based on pigmentation of the primaries [36]. Two age classes were recognized, first year birds (juveniles = juv) and older birds (adults = ad). Spring samples for aging were birds found dead (mostly killed by gyrfalcons), birds trapped for banding or birds photographed while flying using high speed cameras.

#### Age ratios in late Summer (fecundity)

In the last week of July and the first week of August we searched actively on foot for ptarmigan. We distinguished between adults (males and females) and chicks according to size, color, and sound. The age ratio was calculated using total number of females observed and assuming that half of the chicks were females. This ratio was used as a measure of fecundity.

#### Mortality rates

Mortality rates were calculated according to [35]. For these calculations, a year is defined starting on 1^st^ May and ending 30^th^ April. Two mortality rates are recognized: (1) Z_2_ or apparent adult mortality rate; (2) Z_*X*,*W*_ or juvenile excess mortality (mortality that juveniles suffer in excess to adults).

The population abundance index and spring age ratios were used to estimate the Z_2_ mortality rate, assuming that spring abundance was proportional to the total number of birds in the study area. It was assumed that any bird alive at the end of winter was either in its second-year or older at the end of the following winter, provided it survived. So, adult mortality from spring to spring was calculated as:

$${Ẑ}_{2}^{t}=\ln\left(Y^{t-1}\right)-\ln\left(Y^{t}\right)-ln({\hat{p}}_{2}^{t})$$

where

*Y*^*t*^ = spring abundance index year *t*

*Y*^*t*−1^ = spring abundance index year *t* ─ 1

${\hat{p}}_{2}^{t}$ = fraction of adult birds in spring year *t*

The Z_*X*,*W*_ mortality rate describes mortality that first year birds suffer from 1 August to 30 April in excess to adult mortality. The age ratios in late summer and at the end of the following winter were used to estimate excess juvenile mortality as:

$${Ẑ}_{X,W}^{t}=ln\;(\frac{{\hat{p}}_{1}^{t,s}}{{\hat{p}}_{2}^{t,s}})-\ln\;\left(\frac{{\hat{p}}_{1}^{t}}{{\hat{p}}_{2}^{t}}\right)$$

where

${\hat{p}}_{1}^{t,s}$ = fraction of juvenile birds late summer

${\hat{p}}_{1}^{t}$ = fraction of juvenile birds at end of winter

${\hat{p}}_{2}^{t,s}$ = fraction of adult birds late summer

${\hat{p}}_{2}^{t}$ = fraction of adult birds at end of winter

Juveniles share Z_2_ with the adults or at least a rate that shows the same trend [51]. Accordingly, the total mortality rate of juveniles was approximated as the sum of the Z_2_ and the Z_*X*,*W*_ rates.

### Statistical Analysis

We calculated annual prevalence and confidence intervals and discrepancy indices for each parasite species using the software QP web [53]. All other statistical analyses were performed using the software package R [54]. Tests were two-tailed and statistical significance was set at p ≤ 0.05.

We used generalized linear models (GLMs) to assess the relationship between body condition (response variables) and parasite measures (explanatory variables) of individual birds. Gaussian family with identity link was specified to examine parasite richness and abundance of all parasites, ectoparasites, and endoparasites. Binomial family with logit link was specified to examine prevalence of the individual parasite species. Alpha levels (p ≤ 0.05) were adjusted using Holm–Bonferroni corrections.

We used linear regression models to test whether and how trajectories of ptarmigan mortality, fecundity, and population density (response variables) are related with parasite measures (explanatory variables). For density, parasite measures were related with current year ptarmigan densities. The time lag was estimated by fitting regressions with different time lags. The degrees of freedom, n-3, were calculated taking into account the estimation of the time lag in addition to the slope and the intercept. Because ptarmigan density measures are from spring and parasite measures from autumn, there is a 0.5 year time difference between current year parasite numbers and ptarmigan densities, a 1.5 year difference with densities 1 calendar year ago, and a 2.5 year difference with densities 2 calendar years ago. For all models, each age group was examined individually.

## Results

### Ptarmigan Demographics

#### Population density

**Fig 1:**
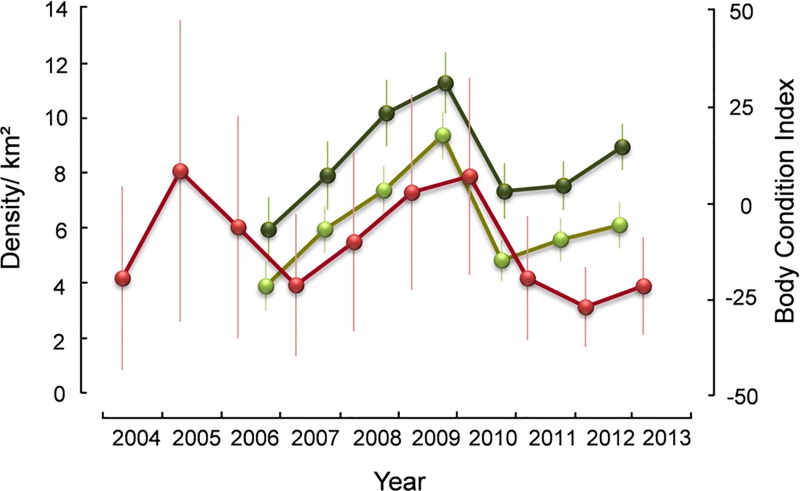
Red line: Mean population densities (± 95% confidence intervals) of male rock ptarmigan on 6 census plots from spring 2004–13 in northeast Iceland. **Green lines: Body condition indices** (± 95% confidence intervals) **of juvenile** (lighter green) **and adult** (darker green) **ptarmigan from autumn 2006–12 in northeast Iceland. **

There were peaks in ptarmigan numbers in 2005 (8.1 cocks/ km²) and 2010 (7.9 cocks/ km²), and lows in 2007 (4.0 cocks/ km²) and 2012 (3.5 cocks/ km²) (Fig 1). During the tenure of our parasite study, the ptarmigan population increased for 4 years and decreased for 3 years (Fig 1).

#### Body condition

Adults were in a better condition than juveniles, but the trajectories showed the same pattern, rising to a peak in 2009, falling sharply in 2010, and improving slightly in 2011 and 2012 (Fig 1).

#### Mortality rates

The Z_2_ mortality rate changed in a regular fashion; it was high at the start of the study in 2005/2006, decreased to 2007/2008, increased to a peak in 2009/2010, and decreased again (Table 1). The Z_*X*,*W*_ mortality rate fluctuated in an irregular fashion over the course of the study (Table 1).

**Table 1:** Mortality rates of rock ptarmigan in northeast Iceland, 2006–12.

Year	Mortality rates		
	Z_2_	Z_*X*,*W*_	Z_2_ + Z_*X*,*W*_
2006/2007	1.29	0.87	2.16
2007/2008	0.77	0.61	1.38
2008/2009	0.47	1.11	1.58
2009/2010	1.08	0.44	1.52
2010/2011	1.40	1.00	2.40
2011/2012	0.92	0.89	1.81
2012/2013	0.84	0.84	1.68

Z_2_ = Annual mortality rate of adults; Z_*X*,*W*_ = Juvenile excess winter mortality; Z_2_ + Z_*X*,*W*_ = Annual mortality rate of juveniles.

#### Fecundity

**Fig 2:**
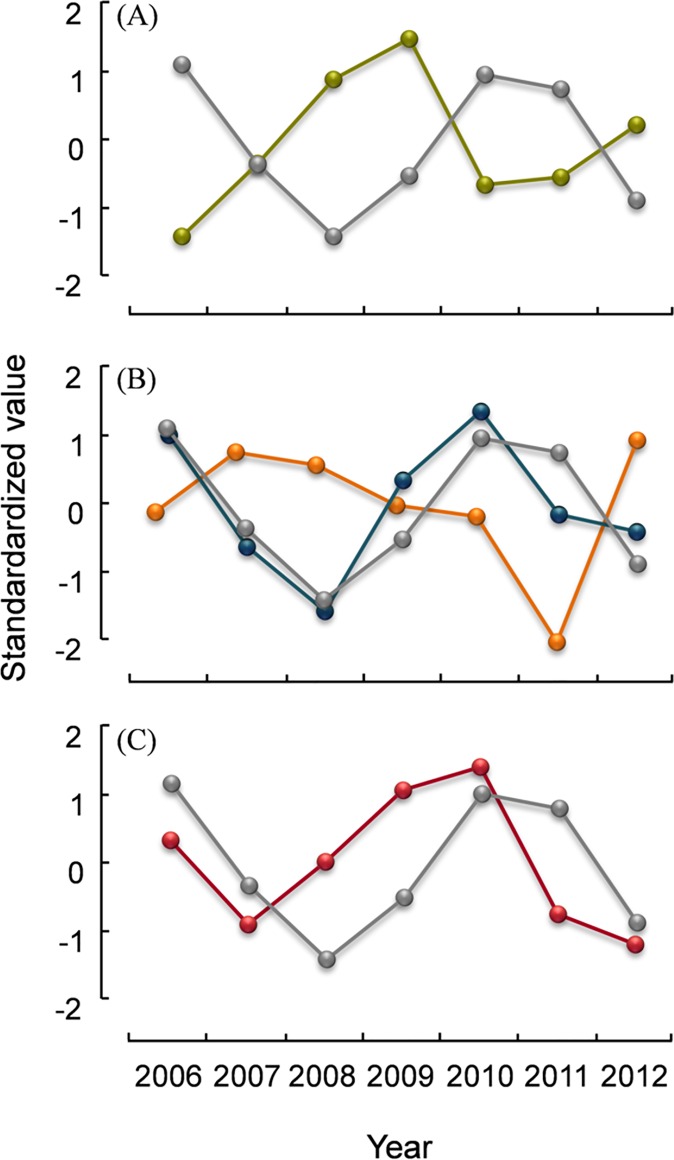
**Trajectories of *Eimeria muta* prevalence** (grey line) **and (A) body condition** (green line) **of adult rock ptarmigan, (B) adult mortality rate Z**_**2**_ (blue line) **and fecundity** (orange line)**, and (C) ptarmigan population density** (red line) **in northeast Iceland, 2006–12.** Values are standardized to (X-μ)/σ.

Fecundity was high all years except in 2011 (Table 2, Fig 2B).

**Table 2:** Age ratios of rock ptarmigan in northeast Iceland, 2006–13. Late summer age ratio is equivalent to Fecundity.

Year	Age ratios	
	Spring (n)	Late summer (n)
2006		0.77 (367)
2007	0.57 (163)	0.79 (318)
2008	0.67 (162)	0.79 (511)
2009	0.55 (155)	0.77 (631)
2010	0.68 (262)	0.76 (550)
2011	0.54 (275)	0.71 (246)
2012	0.49 (292)	0.80 (366)
2013	0.63 (251)	

### Parasites

We examined 631 ptarmigan– 416 juveniles and 215 adults of which 630 (99.7%) had at least one parasite species. The two parasite-free birds were adult males. There were 616 birds (98%) infested with ectoparasites and 536 (85%) infected with endoparasites. Of those, 572 birds (91%) carried mites, 509 (81%) mallophagans, 505 (80%) coccidians, 245 (39%) hippoboscids, 201 (32%) helminths, and 2 (0.3%) fleas. From the six chosen pathogenic parasite species, *E*. *muta* was most prevalent (78%), followed by *C*. *caudinflata* (29%), *M*. *islandicus* (23%), *E*. *rjupa* (15%), *A*. *lagopi* (13%), and *T*. *tenuis* (4%).

#### Combined parasite community

**Fig 3:**
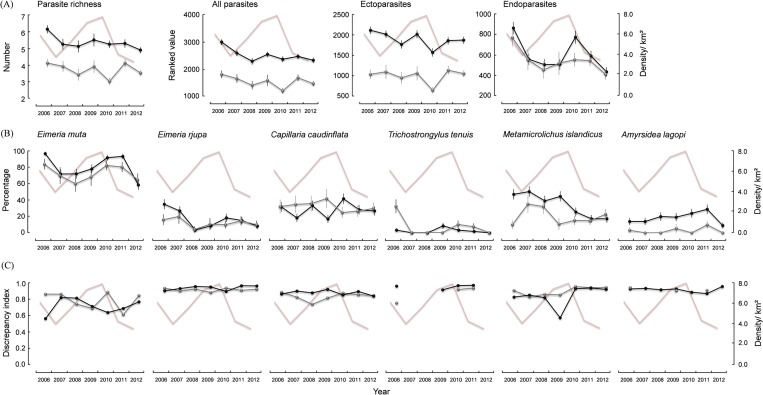
**(A) Parasite species richness** (± SE) **and ranks** (± SE) **for all parasites, ectoparasites, and endoparasites, (B) prevalence** (± SE) **and (C) aggregation of selected pathogenic parasite species of juvenile** (black line) **and adult** (grey line) **rock ptarmigan in northeast Iceland, 2006–12**. Rosy line in background represents ptarmigan population density.

Juveniles carried more parasite species and ectoparasites than adults, but endoparasite numbers were overall similar in both age groups (Fig 3A). Parasite trajectories fluctuated over the years of this study, but were particularly distinct for endoparasites with peaks in 2010 and descents from 2006 to 2008/2009 and from 2010 to 2012 in both age groups (Fig 3A).

#### Individual parasite species

More juveniles than adults carried *E*. *muta*, *E*. *rjupa*, *M*. *islandicus*, and *A*. *lagopi*, but *C*. *caudinflata* and *T*. *tenuis* tended to be more prevalent in adults (Fig 3B). *E*. *muta* and *E*. *rjupa* trajectories fluctuated similarly, with peaks in 2010/ 2011 and lows in 2007/2008 for both age groups (Fig 3B). Prevalence of *M*. *islandicus* in juveniles decreased from 2006 to 2012 interspersed with a peak year in 2007 and a bottom year in 2009 in adults (Fig 3B). Prevalences of *C*. *caudinflata* and *A*. *lagopi* went up and down in every year of this study (Fig 3B).

The parasite species were highly aggregated, and the aggregation index for the different parasite species except for *E*. *muta* changed little over the years of this study (Fig 3C). The aggregation index of *E*. *muta* in juveniles peaked in 2007, reached a low in 2010, and then increased again (Fig 3C).

### Parasites and Ptarmigan Demographics

#### Population density

Endoparasites and in particular *E*. *muta* prevalence showed a significant positive relationship with ptarmigan population density in both age groups. *E*. *muta* aggregation in juveniles showed a significant inverse relationship with density. The best fit provided the model considering a time lag of 1 year (Table 3). That is, endoparasite abundance and *E*. *muta* prevalence peaked and *E*. *muta* aggregation was lowest 1.5 years after the peak in ptarmigan numbers (Figs 2 and 3). Surprisingly, *T*. *tenuis* prevalence in juveniles showed a significant negative relationship with ptarmigan population density, a 2 year time lag fitted best, but prevalence for this species was very low (Table 3).

**Table 3:** Results from linear regression models between spring densities (2004–2012) and selected pathogenic parasites (2006–2012) of adult and juvenile rock ptarmigan in northeast Iceland. Density and parasite trajectories were regressed without (t) and with time lag, shifting the parasite trajectories 1 (t-1) and 2 (t-2) years back in time. Best fits are shown.

		Adult ptarmigan					Juvenile ptarmigan				
Measure	Parasite species	Time lag	Intercept	Slope	*R*^*2*^	p	Time lag	Intercept	Slope	*R*^*2*^	p
Parasite richness		t-1	0.54	1.53	0.38	0.415	t-2	22.28	-3.01	-0.71	0.086
All parasites		t	10.43	-3.2×10^−3^	-0.38	0.411	t-1	-4.47	4.3×10^−3^	0.62	0.154
Ectoparasites		t	12.43	-6.9×10^−3^	-0.68	0.109	t	10.51	-2.6×10^−3^	-0.28	0.545
Endoparasites		t-1	-0.14	1.1×10^−2^	0.80	**0.041**	t-1	0.94	8.7×10^−3^	0.81	**0.036**
Prevalence	*Eimeria muta*	t-1	-6.43	0.17	0.97	**0.001**	t-1	-2.46	0.11	0.91	**0.007**
	*Eimeria rjupa*	t-1	3.83	0.19	0.60	0.165	t-1	4.38	0.11	0.74	0.070
	*Capillaria caudinflata*	t-1	10.59	-0.14	-0.47	0.295	t	3.55	7.1×10^−2^	0.36	0.434
	*Trichostrongylus tenuis*	t-1	5.48	0.10	0.72	0.079	t-2	7.25	-0.44	-0.81	**0.038**
	*Metamicrolichus islandicus*	t-2	4.00	0.11	0.68	0.109	t-2	7.88	-4.9×10^−2^	-0.42	0.358
	*Amyrsidea lagopi*	t-1	5.60	0.23	0.52	0.245	t-1	4.21	0.11	0.45	0.319
Aggregation	*Eimeria muta*	t	4.48	1.34	8.3×10^−2^	0.862	t-1	16.43	-14.12	-0.79	**0.043**

#### Body condition

Body condition of adults was significantly negatively related with parasite richness, parasite abundance, and prevalence of *E*. *muta* and *T*. *tenuis*, but positively with *C*. *caudinflata* prevalence (Table 4). Body condition of juveniles was significantly related with parasite richness, parasite abundance, and all selected pathogenic parasite species except *T*. *tenuis* (Table 5).

**Table 4:** Results from linear regression models between trajectories of parasite measures of pathogenic species and body condition indices, annual adult mortality (Z_2_), and brood size of adult rock ptarmigan in northeast Iceland, 2006–2012.

	Body Condition Index		Annual Mortality (Z_2_)				Fecundity			
**Parasite measure**	t value	p	Intercept	Slope	*R*^*2*^	p	Intercept	Slope	*R*^*2*^	p
Parasite richness	3.00	< 0.001*	1.10	3.6×10^−2^	2.1×10^−3^	0.922	0.87	-2.8×10^−2^	1.5×10^−2^	0.397
All parasites	2.82	< 0.001*	0.00	-3.6×10^−5^	5.3×10^−4^	0.961	0.83	-4.0×10^−5^	7.0×10^−2^	0.567
Ectoparasites	2.71	< 0.001*	1.84	-8.8×10^−4^	0.21	0.306	0.79	-2.2×10^−5^	1.4×10^−2^	0.797
Endoparasites	-2.36	< 0.001*	0.04	1.7×10^−3^	0.38	0.141	0.81	-7.8×10^−5^	8.6×10^−2^	0.523
**Parasite species**										
*Eimeria muta*	-2.90	< 0.001*	-1.08	2.8×10^−2^	0.68	**0.022**	0.92	-2.0×10^−3^	0.40	0.129
*Eimeria rjupa*	-1.09	0.293	0.76	1.7×10^−2^	8.1×10^−2^	0.537	0.78	-1.1×10^−3^	3.4×10^−2^	0.693
*Capillaria caudinflata*	1.54	0.024*	1.58	-1.9×10^−2^	0.12	0.447	0.70	2.1×10^−3^	0.17	0.361
*Trichostrongylus tenuis*	-1.91	0.024*	0.85	1.7×10^−2^	0.40	0.125	0.77	-7.2×10^−4^	7.8×10^−2^	0.543
*Metamicrolichus islandicus*	1.03	0.368	1.46	-2.5×10^−2^	0.64	**0.032**	0.74	1.7×10^−3^	0.31	0.196
*Amyrsidea lagopi*	1.14	0.267	0.93	1.4×10^−2^	2.6×10^−2^	0.730	0.79	-7.1×10^−3^	0.79	**0.008**

* adjusted using Holm-Bonferroni corrections.

**Table 5:** Results from linear regression models between trajectories of parasite measures of pathogenic species and body condition indices, annual juvenile mortality (Z_2_ + Z_*X*,*W*_), juvenile excess winter mortality (Z_*X*,*W*_), and brood size of juvenile rock ptarmigan in northeast Iceland, 2006–2012.

	Body Condition Index		Mortality (Z_2_ + Z_*X*,*W*_)				Excess Winter Mortality (Z_*X*,*W*_)				Fecundity			
**Parasite measure**	t value	p	Intercept	Slope	*R*^*2*^	p	Intercept	Slope	*R*^*2*^	p	Intercept	Slope	*R*^*2*^	p
Parasite richness	-3.84	< 0.001*	-7.1×10^−2^	0.35	0.14	0.405	1.36	-0.10	3.0×10^−2^	0.709	0.87	-2.0×10^−2^	6.4×10^−2^	0.583
All parasites	-3.68	< 0.001*	0.98	3.3×10^−4^	4.6×10^−2^	0.645	1.55	-2.9×10^−4^	0.10	0.499	0.81	-1.6×10^−5^	1.6×10^−2^	0.788
Ectoparasites	3.63	< 0.001*	3.40	-8.5×10^−4^	0.18	0.342	2.32	-7.9×10^−4^	0.40	0.125	0.74	1.5×10^−5^	8.5×10^−3^	0.844
Endoparasites	-3.19	< 0.001*	0.62	1.9×10^−3^	0.68	**0.022**	0.59	3.9×10^−4^	7.0×10^−2^	0.565	0.80	-5.9×10^−5^	9.1×10^−2^	0.510
**Parasite species**														
*Eimeria muta*	-3.88	< 0.001*	0.35	1.8×10^−2^	0.48	0.084	0.58	3.0×10^−3^	3.4×10^−2^	0.691	0.90	-1.6×10^−3^	0.54	0.059
*Eimeria rjupa*	-1.70	0.015*	1.58	1.3×10^−2^	0.15	0.392	0.86	-2.5×10^−3^	1.5×10^−2^	0.792	0.77	-2.7×10^−4^	9.9×10^−3^	0.832
*Capillaria caudinflata*	-1.93	< 0.001*	0.80	3.5×10^−2^	0.68	**0.022**	0.17	2.3×10^−2^	0.78	**0.009**	0.79	6.1×10^−4^	3.0×10^−2^	0.712
*Trichostrongylus tenuis*	-1.34	0.125	1.75	2.1×10^−2^	1.7×10^−2^	0.758	0.92	-4.2×10^−2^	0.33	0.181	0.77	-2.4×10^−3^	5.8×10^−2^	0.603
*Metamicrolichus islandicus*	2.07	< 0.001*	2.05	-7.8×10^−3^	9.1×10^−2^	0.510	1.06	-6.8×10^−3^	0.19	0.335	0.74	8.4×10^−4^	0.16	0.380
*Amyrsidea lagopi*	2.41	< 0.001*	1.51	1.6×10^−2^	8.2×10^−2^	0.534	0.67	8.7×10^−3^	6.6×10^−2^	0.577	0.83	-3.7×10^−3^	0.68	**0.023**

* adjusted using Holm-Bonferroni corrections.

#### Mortality rates

**Fig 4:**
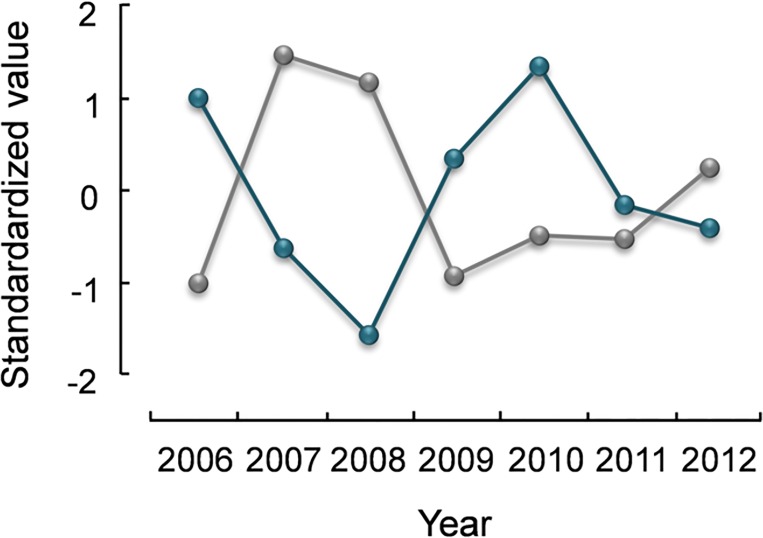
Trajectories of mortality Z_2_ (blue line) and *Metamicrolichus islandicus* prevalence (grey line) of adult rock ptarmigan in northeast Iceland, 2006–12. Values are standardized to (X-μ)/σ.

**Fig 5:**
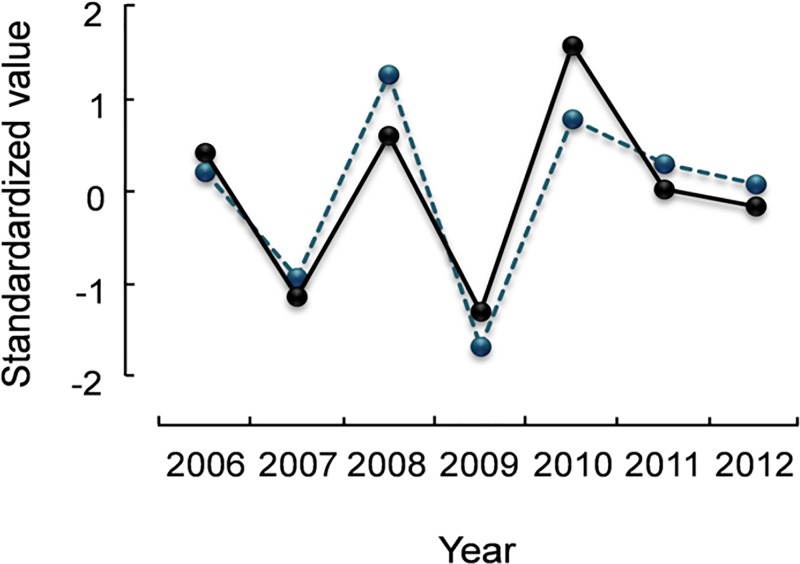
Trajectories of excess winter mortality Z_*X*,*W*_ (blue dashed line) and *Capillaria caudinflata* prevalence (black continuous line) of juvenile rock ptarmigan in northeast Iceland, 2006–12. Values are standardized to (X-μ)/σ.

Annual adult mortality was significantly positively related with prevalence of *E*. *muta* and negatively with *M*. *islandicus* (Table 4, Fig 4). Annual juvenile mortality was significantly positively related with endoparasites and *C*. *caudinflata* prevalence, and marginally with *E*. *muta* prevalence (Table 5). Excess juvenile winter mortality was significantly positively related with *C*. *caudinflata* prevalence (Table 5, Fig 5).

#### Fecundity

**Fig 6:**
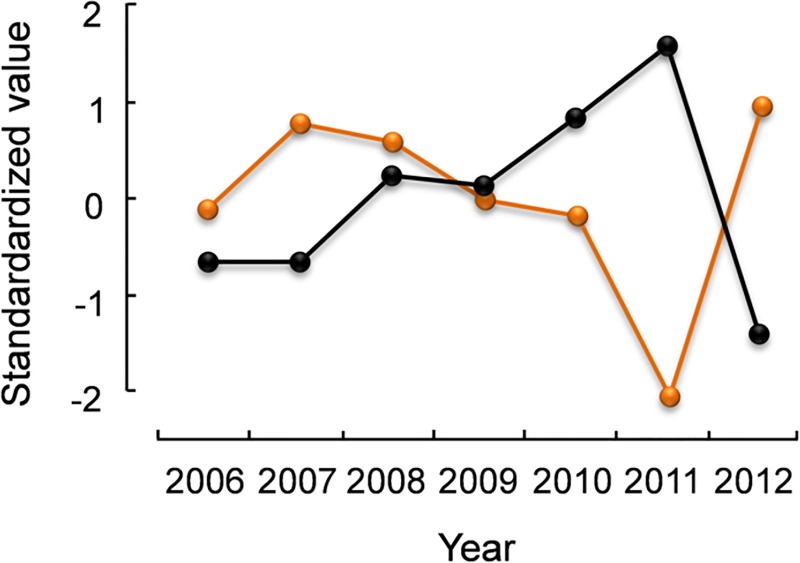
Trajectories of fecundity (orange line) and *Amyrsidea lagopi* prevalence (black line) of juvenile rock ptarmigan in northeast Iceland, 2006–12. Values are standardized to (X-μ)/σ.

Fecundity was significantly positively related with prevalence of *A*. *lagopi* in both age groups and marginally with *E*. *muta* prevalence in juvenile birds (Tables 4 and 5, Fig 6).

## Discussion

The Anderson and May model [4] [31] identified low aggregation of parasites within the host population, parasite-induced host mortality and reduction in host reproductive potential, respectively, as well as time delays in parasite reproduction and transmission as the major regulating or destabilizing qualities that parasites can have on host population dynamics. Our results indicate that the eimerid *E*. *muta* parasitizing rock ptarmigan in Iceland fulfils all of these conditions. Aggregation levels of *E*. *muta* fluctuate inversely with its prevalence, with low aggregation at peak prevalence and vice versa, and both prevalence and aggregation track ptarmigan density with a 1.5 year time lag, respectively. Further, *E*. *muta* is associated with poorer body condition, increased mortality, and reduced fecundity (Fig 2). For red grouse in Scotland, coupled parasite-host cycles have been described and tested experimentally [55] [56]. In this grouse population exhibiting 4–8 year cycles, *Trichostrongylus tenuis* reduced fecundity and survival and there was a density dependent relationship between grouse numbers in one year and worm burdens in the subsequent year [55]. The fairly low levels of parasite aggregation observed in this system increased the tendency of the system to oscillate [55]. Alternatively, ptarmigan body condition after the peak in abundance nosedives for other reasons than parasites making the birds more susceptible to parasitism. Here, the chicken versus the egg question arises: are ptarmigan in poor body condition more susceptible to *Eimeria* or does the parasite cause ptarmigan to be in poor body condition? The Anderson-May model suggests for parasites to have a regulating effect on host numbers, it needs the parasite to affect the host which is what our data implies for *E*. *muta*. However, our study is correlational and so we do not know if *E*. *muta* is sufficiently virulent in itself or if it becomes virulent only or increasingly when the system is already enfeebled due to other factors, or possibly due to the combined parasite community. Parasite richness, the overall parasite community, and majority of chosen pathogenic parasites were all correlated with ptarmigan condition directly (Tables 4 and 5). Whatever the initial trigger may be, our data implies that there is a strong relation between ptarmigan condition, population density, and particularly the eimerid *E*. *muta*.

Ptarmigan eimerids have a direct life cycle with oocysts shed in feces, sporulation in the environment, and infection through ingestion. The oocysts are present in the feces year round, but prevalence of *E*. *muta* peak between October and January [33]. Eimerids generally are host specific [57] and we assume *E*. *muta* (and also *Eimeria rjupa*) will not persist in species other than rock ptarmigan. We know the prepatent period of *Eimeria* varies between 4 and 6 days with a peak in oocyst shedding within 10 days [27] [58]. Thus, oocysts shed by ptarmigan in the first week of October are caused by infections occurring by mid September when the ptarmigan are moving to autumn habitats in alpine areas. Host density-dependent shedding of oocysts and their subsequent persistence in the environment from one year to the next could be the reason for the observed time-lag between ptarmigan numbers and *E*. *muta* prevalence. Ptarmigan hatched in the two years succeeding the peak in their numbers should be exposed to the maximum number of infective oocysts in the environment. Environmental persistence is well known for *Eimeria*, infective oocysts of some species can survive up to 602 days in soil [59], stand repeated freeze and thaw cycles [60], and this life history characteristic is sufficient to maintain eimerid populations [61]. Life history characteristics of parasites including the time needed for helminth larval stages to grow to maturity after infection and arrested development of the helminth larvae, have been given as explanations for time lags between host and parasite populations in similar systems [55] [62].

In addition to the severalfold relationship between population parameters and *E*. *muta*, we found a close relationship between juvenile excess winter mortality and annual juvenile mortality (a measure that partly consists of excess winter mortality) as well as *C*. *caudinflata* prevalence (Fig 5). *Capillaria* species are known to cause severe symptoms such as diarrhoea, weakness, weight loss, and a drop in egg production (capillariasis) [3]. *C*. *caudinflata* has an indirect life-cycle with earthworms as intermediate host [63]. Ptarmigan chicks have a mixed diet of plants and invertebrates such as earthworms, but adult birds eat mainly plants [64]. It is of interest that adult birds in our sample show *C*. *caudinflata* infections. The life span of *C*. *caudinflata* is c. 10 months [65]. This suggests that some of the adults could be carrying infections acquired as juveniles, but high prevalence among adults suggests that also members of this cohort get infected. In Icelandic ptarmigan, prevalence of *C*. *caudinflata* eggs in feces peaks in October and January [33], suggesting peaks of intestinal worm burden and/or worm reproduction during these times of the year. There is an approximate four-month time difference between ptarmigan hatch and the first peak in *C*. *caudinflata* egg shedding in October. The data suggest that juveniles suffer more from *C*. *caudinflata* infections than adults, i.e. the relationship with the mortality rates; *C*. *caudinflata* infections seem to be one of the drivers of the Z_*X*,*W*_ rate (mortality in August through April). That juveniles suffer more from *C*. *caudinflata* infections than adults may have to do with varying levels of immune function to resist or treat infections [66].

The reverse relation between fecundity and the amblyceran chewing louse *Amyrsidea lagopi* prevalence is of interest (Fig 6). It is well known that feather lice can be severely damaging to their host (summaries of various studies in [67]), including by reducing fecundity [68] [69] [70]. The amblyceran mallophagan *Menacanthus stramineus* in high intensities, for instance, was related with reduced egg production [71]]. Contrary in other studies, controlled experiments did not show any effects of lice on reproductive success of swifts *Apus apus* and rock doves *Columba livia* [72] [73]. In Icelandic ptarmigan, *A*. *lagopi* is strongly associated with the creation of feather holes [30]. If this association happens through trade-offs between host reproduction and self-maintenance or for other reasons needs to be tested experimentally. A study on collared flycatchers *Ficedula albicollis* showed that feather wear of parents are traded-off by parental activity, and that the degree of feather wear was associated with survival of the flycatchers [74].

The reverse relation between adult mortality and prevalence of *Metamicrolichus islandicus* is peculiar. When mortality is high, then fewer birds in the population are infested with this parasite that can cause mange. We do not have an explanation for this.

The helminth *Trichostrongylus tenuis* is a pathogenic nematode that was associated with reduced body condition in willow ptarmigan in Norway [12] and has been shown to be the determinant of the red grouse cycle [56]. In our study, this nematode occurred in such low numbers (≤ 28 worms) that it seems improbable that this parasite could have caused serious harm [29]. Yet, this parasite did show an inverse relation with body condition in adult ptarmigan and the 1.5 year time lag is near significant for both age groups.

The combined parasite community correlated with ptarmigan condition, but only endoparasites were also positively correlated with annual mortality in juveniles, probably mostly attributable to *E*. *muta*. Comparatively, though none of the parasites in Norwegian willow ptarmigan had a significant impact on their own, the parasite community was negatively related with host fitness, and this in turn was suggested to promote effects on host body mass and breeding mortality [12].

Population size of the main resident predator, the gyrfalcon, changes also in a delayed density dependent manner with the ptarmigan population which has been suggested to be the main driver of the ptarmigan population cycles [18]. So, parasitism in the ptarmigan population may act directly and/or sublethal parasitism may act synergistically with predation by acting upon different population parameters [63] or by making the ptarmigan more prone to gyrfalcon predation [24] [75] [76].

## Supporting Information

S1 File Data provision. (CSV)
